# Contralateral-controlled thigh muscle biomarkers on CT angiography for prediction of 1-year restenosis after endovascular therapy for unilateral peripheral artery disease

**DOI:** 10.1186/s13244-026-02407-0

**Published:** 2026-10-07

**Authors:** Shuangxiang Lin, Xinhong Wang, Yifeng Pan, Libing Zhang, Jianzhong Sun, Bing Chen

**Affiliations:** 1 Department of Radiology, The Second Affiliated Hospital Zhejiang University School of Medicine, Hangzhou, People’s Republic of China; 2 Department of Vascular Surgery, The Second Affiliated Hospital Zhejiang University School of Medicine, Hangzhou, People’s Republic of China

**Keywords:** Peripheral arterial disease, Computed tomography angiography, Muscle, skeletal, Constriction, pathologic, Endovascular procedures

## Abstract

**Objective:**

To develop and internally validate a model using contralateral-controlled thigh muscle biomarkers, measured on routine computed tomography angiography (CTA), to predict clinically driven, DSA-confirmed 1-year restenosis after endovascular therapy for unilateral peripheral artery disease (PAD).

**Materials and methods:**

In this retrospective cohort study, 378 patients with unilateral PAD undergoing endovascular therapy and having baseline and 1-year follow-up CTA were included. Mid-thigh skeletal muscle cross-sectional area and whole-thigh muscle volume were quantified bilaterally with semi-automated software. Contralateral-controlled biomarkers (affected minus contralateral) were derived to isolate limb-specific ischemic effects. The primary endpoint was clinically driven 1-year restenosis (≥ 50%) confirmed by DSA.

**Results:**

Among 378 patients, 162 (43%) developed restenosis at a median follow-up of 12.5 months. Contralateral-controlled muscle asymmetry was independently associated with restenosis after adjustment for lesion length, baseline stenosis degree, Fontaine stage, disease duration, and TASC II classification (OR 1.43, 95% CI: 1.08–1.89, *p* = 0.01). Adding muscle asymmetry to a severity-only model improved discrimination from AUC 0.76 to 0.79 (DeLong *p* = 0.04). A baseline predictive model combining lesion length and muscle asymmetry achieved AUC 0.74; a full explanatory model incorporating longitudinal muscle changes reached AUC 0.88, with the greatest net benefit on decision curve analysis.

**Conclusion:**

Thigh muscle biomarkers, acquired opportunistically from routine CTA scans, improved the prediction of 1-year restenosis after endovascular therapy and may support targeted surveillance in patients with unilateral PAD.

**Key Points:**

**Question** Can contralateral-controlled thigh muscle biomarkers on routine CT angiography improve the prediction of 1-year restenosis after endovascular therapy for unilateral peripheral artery disease?

**Findings** In 378 PAD patients, baseline contralateral thigh muscle asymmetry independently predicted clinically driven, DSA-confirmed restenosis and improved AUC from 0.76 to 0.79.

**Critical relevance statement** Opportunistic contralateral-controlled thigh muscle analysis on routine CT angiography provides a no-cost imaging biomarker that refines post-endovascular restenosis risk stratification, supporting targeted angiographic surveillance and advancing personalized vascular imaging practice in peripheral artery disease.

**Graphical Abstract:**

**Figure:**
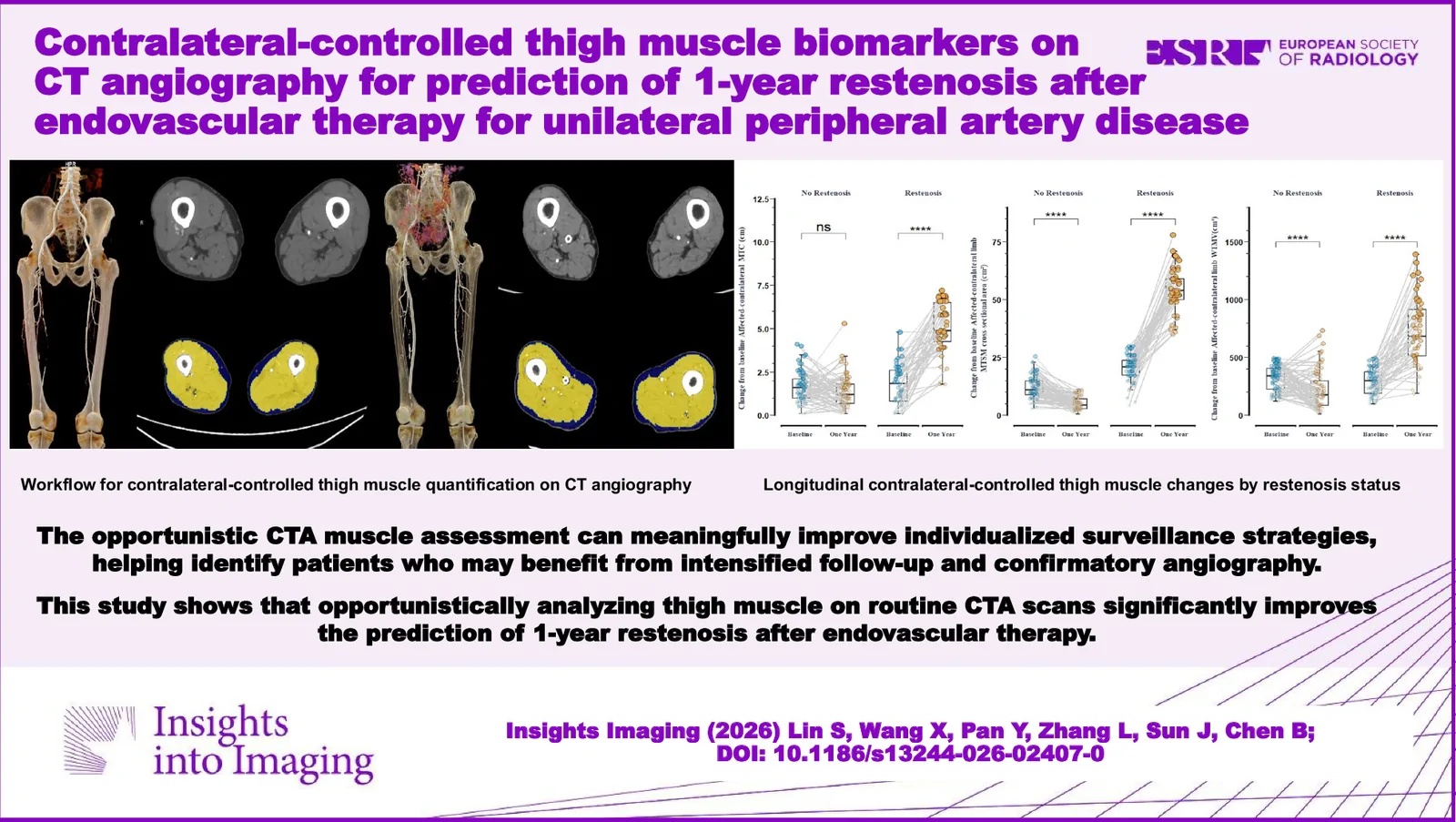

## Introduction

Peripheral artery disease (PAD), a prevalent form of systemic atherosclerosis affecting about 200 million people globally, is a leading cause of impaired mobility and chronic limb-threatening ischemia [1, 2]. Revascularization is often necessary, but its long-term success is limited by restenosis, with in-stent restenosis occurring in 20–40% of patients within 1 year [3]. Symptoms such as claudication, rest pain, and tissue loss severely impair quality of life [4]. While clinical assessment of treatment success relies on symptoms, walk tests, blood pressure measurements, and Doppler sonography, lower-extremity CT angiography is the standard noninvasive imaging tool for detailed anatomical assessment. As these scans are already routine, they enable the opportunistic screening for sarcopenia and quantification of thigh muscle composition without additional imaging burden [5].

CT-based studies have long shown that PAD is accompanied by adverse leg muscle phenotypes and that these phenotypes carry prognostic value: lower calf muscle density and greater fatty infiltration on CT have been associated with higher mortality in PAD [6, 7]. Early clinical and imaging work established that PAD is accompanied by adverse leg muscle phenotypes and that these phenotypes carry prognostic information beyond the arterial lesion [8]. Using CT-based measures of calf muscle composition, Pei-Shan Tsai reported that using CT-based quantification, that the severity of lower-extremity PAD is directly associated with region-specific muscle wasting in the legs, which occurs earlier than central muscle loss [9]. Subsequent studies extended these observations to broader adverse events, showing that CT-derived leg muscle characteristics, particularly low muscle density, were associated with higher risks of cardiovascular and limb events in PAD [10]. More recently, observational analyses and reviews have reinforced sarcopenia as a prognostic factor for mortality and major limb outcomes in chronic limb-threatening ischemia, emphasizing that muscle depletion reflects systemic frailty and limb end-organ compromise [11, 12].

However, most prior investigations have been cross-sectional, have focused on mortality or major limb outcomes rather than device durability, or have relied on single-limb absolute muscle measures that are vulnerable to between-patient confounding from body habitus, metabolic status, and physical activity [13]. Longitudinal evidence linking pre-treatment muscle composition and post-treatment muscle remodeling to angiographically confirmed 1-year restenosis after contemporary endovascular therapy remains limited, and few studies have systematically leveraged the contralateral limb as an internal control to isolate local, limb-specific end-organ signatures of ischemia.

Therefore, the aim of our study was to develop and internally validate a CTA-based risk stratification model for predicting clinically driven, DSA-confirmed 1-year restenosis after balloon angioplasty or stent implantation in unilateral lower-extremity PAD by using contralateral-controlled thigh muscle metrics. We hypothesized that lower contralateral-controlled muscle quantity and poorer muscle quality at baseline, as well as unfavorable longitudinal muscle remodeling, would be independently associated with a higher risk of restenosis beyond traditional clinical risk factors and procedural category, enabling more targeted selection of patients for confirmatory angiography.

## Materials and methods

### Study population

This single-center retrospective cohort study was approved by the institutional review board (I20251118), and the requirement for informed consent was waived for this retrospective analysis of data collected for clinical purposes. Consecutive patients with symptomatic lower-extremity peripheral artery disease who underwent evaluation at our institution between January 2019 and December 2024 were screened. Patients were eligible if they had PAD confirmed by symptoms and CT angiography, had atherosclerotic lesions involving the iliac, femoral, or popliteal segments on baseline CTA, had arteriosclerosis obliterans with Fontaine stage II–IV symptoms, underwent first-time endovascular therapy of the target limb with balloon angioplasty or stent implantation, and had both baseline CTA and approximately 1-year follow-up CTA available for analysis.

**Fig. 1:**
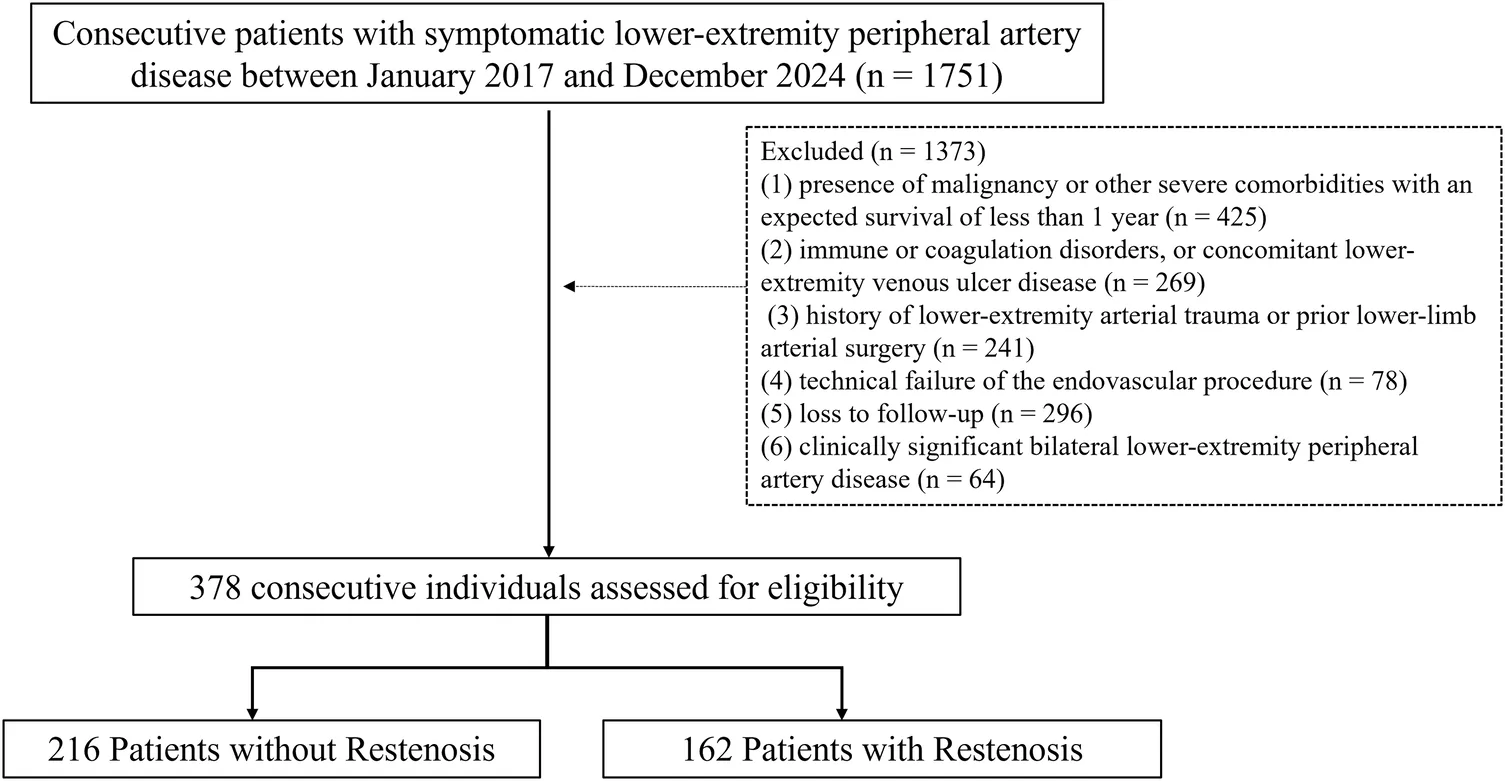
Flowchart of study population

The exclusion criteria were as follows: (1) presence of malignancy or other severe comorbidities with an expected survival of less than 1 year; (2) immune or coagulation disorders, or concomitant lower-extremity venous ulcer disease; (3) history of lower-extremity arterial trauma or prior lower-limb arterial surgery; (4) technical failure of the endovascular procedure; (5) loss to follow-up; and (6) clinically significant bilateral lower-extremity peripheral artery disease, defined as ≥ 50% diameter stenosis in any segment of the contralateral limb on baseline CTA. This criterion was applied to enable contralateral-limb control, ensuring that each included patient had a clearly defined affected limb and a hemodynamically normal contralateral limb (confirmed by CTA showing < 50% stenosis in all iliac, femoral, and popliteal segments and by ankle-brachial index ≥ 0.9) serving as an internal reference. Figure 1 presents the subject flowchart for the study.

### Baseline disease severity assessment

To characterize disease burden beyond lesion length, we recorded the following baseline severity measures for each patient: (1) degree of baseline stenosis, quantified as the maximum percent diameter stenosis of the target lesion on CTA and categorized as 50–69%, 70–89%, or 90–99% occlusion; (2) Fontaine clinical stage (II, III, or IV); (3) estimated disease duration, defined as the interval in months from first symptom onset to the date of endovascular intervention; and (4) TASC II lesion classification (A, B, C, or D). These variables were included in sensitivity analyses to assess whether muscle metrics provided independent prognostic value beyond conventional disease severity measures.

### Imaging protocol

CTA was performed on a multidetector CT system (SOMATOM Definition Flash or SOMATOM Force, Siemens Healthineers, Forchheim, Germany) with patients in the supine position using a standard lower-extremity run-off protocol to cover the bilateral arterial tree. The scan range was from the T12 to the toes. Bolus tracking was used to trigger the scan, with the trigger point located at the abdominal aorta. A region of interest (ROI) was placed in the abdominal aorta, with a threshold of 210 HU. Once this threshold was reached, the scan was automatically initiated after a 10-s delay. Contrast enhancement was achieved with 120 mL of iodinated contrast material (Omnipaque 350, GE Healthcare, USA) injected at 4–5 mL/s, followed by a 60 mL saline flush. Technical parameters were as follows: tube voltage 70–100 kV, effective tube current 400–900 mAs, gantry rotation time 0.25–0.50 s, pitch 0.7–0.9, and collimation 256 × 0.625 mm or 192 × 0.6 mm. Images were reconstructed at 1.0-mm slice thickness with a 1-mm increment using a Bv36 kernel and iterative reconstruction (ADMIRE, strength 4).

### CT image analysis and thigh muscle composition metrics

**Fig. 2:**
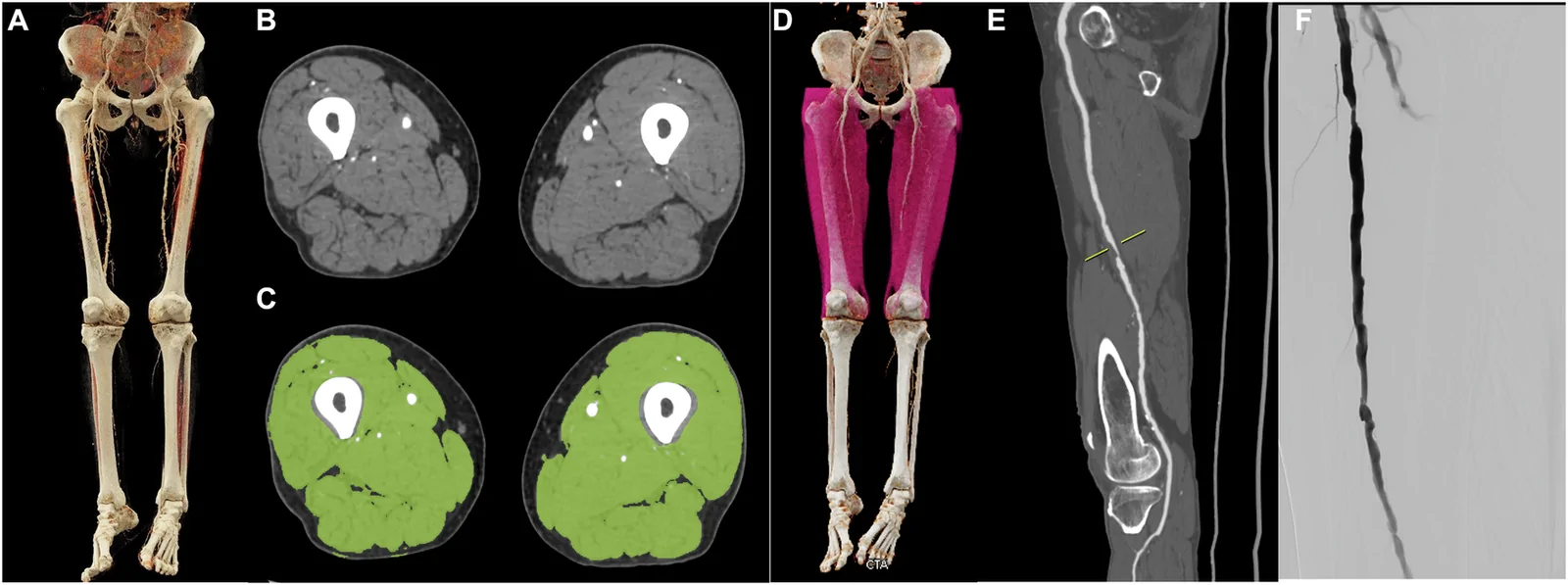
Workflow of contralateral-controlled thigh muscle quantification on CTA. **A** Volume-rendered CTA image of the lower extremity. **B** Axial CTA at a standardized mid-thigh level, showing the affected and contralateral thighs. **C** Thresholding (−29 to 150 HU) for skeletal muscle identification (yellow-green). **D** Muscle segmentation mask overlaid (yellow) to derive the mid-thigh skeletal muscle cross-sectional area. **E** 3D volumetric reconstruction of whole-thigh muscle. **F** Corresponding lesion on DSA

All CTA datasets were imported into 3D Slicer (version 5.8.1), an open-source medical image computing and visualization platform, and processed for quantitative analysis. Two radiology residents (with 3 and 4 years of experience, respectively), blinded to angiographic outcomes, independently performed the measurements. At a standardized mid-thigh level, mid-thigh circumference (MTC) was obtained by tracing the outer skin contour to compute the cross-sectional perimeter, and mid-thigh skeletal muscle (MTSK) was recorded as the skeletal muscle cross-sectional area after muscle segmentation using established CT attenuation thresholds for skeletal muscle of −29 to 150 HU [14]. Whole-thigh muscle volume (WTMV) was derived by segmenting skeletal muscle throughout the predefined thigh segment on consecutive axial slices and aggregating slice-wise muscle masks to obtain a volumetric measure. To reduce between-patient confounding related to body habitus and systemic factors, we derived contralateral-controlled features including side-to-side differences in thigh muscle metrics, expressed as the magnitude of the affected-contralateral difference at each time point, and differential longitudinal change metrics, calculated as the 1-year change in the affected limb minus the 1-year change in the contralateral limb. Figure 2 presents the measurement procedures of the patients. Supplementary Materials case 1 and 2 present the specific cases.

### Follow-up and restenosis assessment

Patients underwent standardized outpatient follow-up after endovascular therapy according to the institutional clinical pathway. Follow-up assessment consisted of interval history for recurrent or worsening ischemic symptoms, including return of claudication, new rest pain, delayed wound healing, or new/non-healing ulcers, together with clinical examination of limb status (including skin and wound inspection, ischemic changes, and lower-extremity pulse assessment) and measurement of resting ankle-brachial index (ABI) of the treated limb, with a clinically meaningful decrease from the post-procedural baseline (typically a drop of ≥ 0.15) considered a trigger for further imaging. Additional vascular evaluation was performed when symptoms recurred or worsened, when limb findings deteriorated, when ABI declined meaningfully, or when restenosis was clinically suspected by the treating physician. Duplex ultrasound was not performed as a universal surveillance test in all patients; rather, it was selectively performed in clinically suspicious cases, using a peak systolic velocity ratio (PSVR) > 2.4 as the threshold suggestive of ≥ 50% stenosis. Digital subtraction angiography (DSA) was performed when clinically indicated by recurrence of ischemic symptoms, clinical deterioration at follow-up, or abnormal duplex ultrasound findings (PSVR > 2.4). All patients also underwent approximately 1-year follow-up CTA (median 12.5 months), which in the present study was used for longitudinal thigh muscle quantification and not as the reference standard for the primary endpoint. The primary endpoint was clinically driven 1-year restenosis confirmed by DSA, defined as at least 50% diameter stenosis within the treated segment; for stented segments, restenosis included luminal re-narrowing within the stent and within 5 mm proximal and distal to the stent edges.

### Statistical analysis

Statistical analyses were performed using R version 4.0.5 (R Foundation for Statistical Computing, Vienna, Austria). Continuous variables are presented as mean ± standard deviation and categorical variables as counts and percentages. Normality was assessed using the Shapiro–Wilk test. Between-group comparisons were performed using the independent-samples *T*-test or Mann–Whitney U test for continuous variables and the chi-square test or Fisher’s exact test for categorical variables; All tests were two-sided, and a *p*-value < 0.05 was considered statistically significant. For risk stratification, a series of multivariable logistic regression models was developed. To address the utility of the model for both prediction and explanation, we constructed the models in a stepwise fashion. Model 1, a basic clinical model, included only lesion length. Model 2, our primary predictive model, added the prespecified baseline contralateral-controlled muscle metric. Model 3, an exploratory/explanatory model, incorporated post-procedural (1-year) muscle metrics to assess the impact of longitudinal changes. Candidate predictors were prespecified based on clinical relevance. When two variables were highly correlated (mid-thigh skeletal muscle area and volume), the one with the stronger univariable association with the outcome was retained for multivariable modeling to enhance model stability. The number of events per predictor variable was calculated for each model to assess the risk of overfitting. Model performance was evaluated in terms of discrimination and clinical utility. Discrimination was quantified using the area under the receiver operating characteristic curve with 95% confidence intervals, with internal validation performed via 2000-repetition bootstrapping to correct for optimism. Clinical utility for the prespecified decision of performing confirmatory DSA was assessed using decision curve analysis by comparing net benefit across a range of threshold probabilities against the default strategies of performing DSA in all patients or in no patients. For quantitative variables, the intraclass correlation coefficient (ICC) and its 95% confidence interval (CI) were used to assess reliability.

## Results

### Baseline characteristics

With a median follow-up of 12.5 months, restenosis occurred in 162 of the 378 patients, while the remaining 216 did not develop restenosis. As summarized in Table 1, compared with the no-restenosis group, the restenosis group was taller (163.35 ± 7.31 cm vs 159.75 ± 6.68 cm, *p* = 0.01) and had longer lesions (6.53 ± 1.85 cm vs 2.19 ± 1.80 cm, *p* < 0.01). Regarding disease severity, the restenosis group had a higher mean baseline stenosis degree (82.4 ± 11.3% vs 71.6 ± 14.7%, *p* < 0.01), a greater proportion of Fontaine stage III–IV disease (62.3% vs 38.4%, *p* < 0.01), longer disease duration (18.7 ± 12.3 months vs 11.2 ± 8.9 months, *p* < 0.01), and more advanced TASC II classification (TASC C/D: 58.6% vs 31.9%, *p* < 0.01). At baseline, patients with restenosis had a smaller affected-limb mid-thigh circumference (40.88 ± 5.13 cm vs 44.23 ± 8.53 cm, *p* = 0.01), a smaller affected-limb mid-thigh skeletal muscle area (176.14 ± 49.32 cm² vs 207.83 ± 47.96 cm², *p* < 0.01), and a larger affected-minus-contralateral difference in mid-thigh skeletal muscle area (20.50 ± 5.67 cm² vs 11.89 ± 4.74 cm², *p* < 0.01). Affected limb ankle-brachial index (ABI) was lower in the restenosis group (0.44 ± 0.17 vs 0.58 ± 0.16, *p* < 0.01), whereas contralateral limb ABI was normal in both groups (1.02 ± 0.12 vs 0.98 ± 0.14, *p* = 0.08), confirming hemodynamic normality of the reference limb.

**Table 1:** Baseline characteristics of the study population

Characteristics	Total	No restenosis (*n* = 216)	Restenosis	*p*-value
	(*n* = 378)		(*n* = 162)	
Age (years)	70.00 ± 6.48	70.19 ± 6.13	69.74 ± 6.97	0.7
Gender (male, %)	183 (48.41)	108 (50)	75 (46.3)	0.68
Height (cm)	161.29 ± 7.15	159.75 ± 6.68	163.35 ± 7.31	0.01
Weight (kg)	55.85 ± 6.65	55.21 ± 5.42	56.70 ± 7.98	0.24
BMI	21.41 ± 1.59	21.62 ± 1.50	21.14 ± 1.68	0.1
Hypertension (%)	198 (52.38)	114 (52.78)	84 (51.85)	0.92
Dyslipidemia (%)	225 (59.52)	126 (58.33)	99 (61.11)	0.75
Diabetes (%)	276 (73.02)	162 (75)	114 (70.37)	0.56
Smoking history (%)	225 (59.52)	126 (58.33)	99 (61.11)	0.75
Disease severity				
Disease duration (months)	14.4 ± 11.2	11.2 ± 8.9	18.7 ± 12.3	< 0.01
Fontaine stage (%)				< 0.01
Stage II	195 (51.59)	133 (61.57)	62 (38.27)	
Stage III	124 (32.80)	64 (29.63)	60 (37.04)	
Stage IV	59 (15.61)	19 (8.80)	40 (24.69)	
Baseline stenosis degree (%)	76.2 ± 13.8	71.6 ± 14.7	82.4 ± 11.3	< 0.01
Stenosis category (%)				< 0.01
50–69%	105 (27.78)	79 (36.57)	26 (16.05)	
70–89%	171 (45.24)	99 (45.83)	72 (44.44)	
90–99%	102 (26.98)	38 (17.59)	64 (39.51)	
TASC II classification (%)				< 0.01
A	78 (20.63)	60 (27.78)	18 (11.11)	
B	128 (33.86)	87 (40.28)	41 (25.31)	
C	108 (28.57)	47 (21.76)	61 (37.65)	
D	64 (16.93)	22 (10.19)	42 (25.93)	
Affected limb ABI	0.52 ± 0.18	0.58 ± 0.16	0.44 ± 0.17	< 0.01
Contralateral limb ABI	1.00 ± 0.13	1.02 ± 0.12	0.98 ± 0.14	0.08
Treatment (%)				0.92
Drug balloon	177 (46.83)	102 (47.22)	75 (46.3)	
STENT	201 (53.17)	114 (52.78)	87 (53.7)	
Length of the lesion (cm)	4.36 ± 1.83	2.19 ± 1.80	6.53 ± 1.85	< 0.01
Baseline				
Affected limb MTC (cm)	42.80 ± 7.43	44.23 ± 8.53	40.88 ± 5.13	0.01
Contralateral limb MTC (cm)	43.32 ± 6.80	43.84 ± 7.63	42.64 ± 5.50	0.33
Affected-contralateral MTC (cm)	1.68 ± 1.01	1.63 ± 0.89	1.75 ± 1.15	0.52
Affected limb MTSM cross-sectional area (cm²)	194.25 ± 50.85	207.83 ± 47.96	176.14 ± 49.32	< 0.01
Contralateral limb MTSM cross-sectional area (cm²)	196.09 ± 49.15	195.67 ± 48.40	196.65 ± 50.60	0.91
Affected-contralateral limb MTSM cross-sectional area (cm²)	15.58 ± 6.68	11.89 ± 4.74	20.50 ± 5.67	< 0.01
Affected limb WTMV (cm³)	7133.46 ± 1691.57	7204.76 ± 1666.13	7038.40 ± 1736.00	0.59
Contralateral limb WTMV(cm³)	7074.84 ± 1720.22	6881.98 ± 1671.13	7331.97 ± 1766.38	0.15
Affected-contralateral limb WTMV(cm³)	310.26 ± 111.20	322.78 ± 109.19	293.57 ± 112.67	0.15
1-year follow-up				
Affected limb MTC (cm)	37.90 ± 8.96	43.24 ± 4.87	30.79 ± 8.22	< 0.01
Contralateral limb MTC (cm)	40.61 ± 7.84	44.16 ± 4.62	35.88 ± 8.74	< 0.01
Affected-contralateral MTC (cm)	2.96 ± 2.18	1.36 ± 0.92	5.09 ± 1.41	< 0.01
Affected limb MTSM cross-sectional area (cm²)	203.41 ± 51.32	194.58 ± 49.58	215.18 ± 51.69	0.03
Contralateral limb MTSM cross-sectional area (cm²)	223.12 ± 65.35	188.04 ± 47.29	269.90 ± 56.35	< 0.01
Affected-contralateral limb MTSM cross-sectional area (cm²)	26.38 ± 25.45	5.13 ± 2.79	54.72 ± 9.27	< 0.01
Affected limb WTMV (cm³)	4352.08 ± 1527.18	4434.09 ± 1770.96	4290.57 ± 1325.14	0.62
Contralateral limb WTMV (cm³)	4539.10 ± 1651.37	4068.68 ± 1314.75	5166.33 ± 1847.85	< 0.01
Affected-contralateral limb WTMV (cm³)	440.61 ± 337.97	221.89 ± 166.38	732.24 ± 284.03	< 0.01
Change from baseline				
Affected-contralateral MTC (cm)	−3.58 ± 9.85	−0.37 ± 1.90	−7.86 ± 13.83	< 0.01
Affected-contralateral limb MTSM cross-sectional area (cm²)	−0.64 ± 1.98	0.49 ± 0.31	−2.16 ± 2.25	< 0.01
Affected-contralateral limb WTMV (cm³)	−0.70 ± 1.68	0.22 ± 0.59	−1.93 ± 1.86	< 0.01

Variables are presented as median (interquartile range) or number (%)

*MTC* mid-thigh circumference, *MTSK* mid-thigh skeletal muscle, *WTMV* whole-thigh muscle volume

### Follow-up and longitudinal changes

**Fig. 3:**
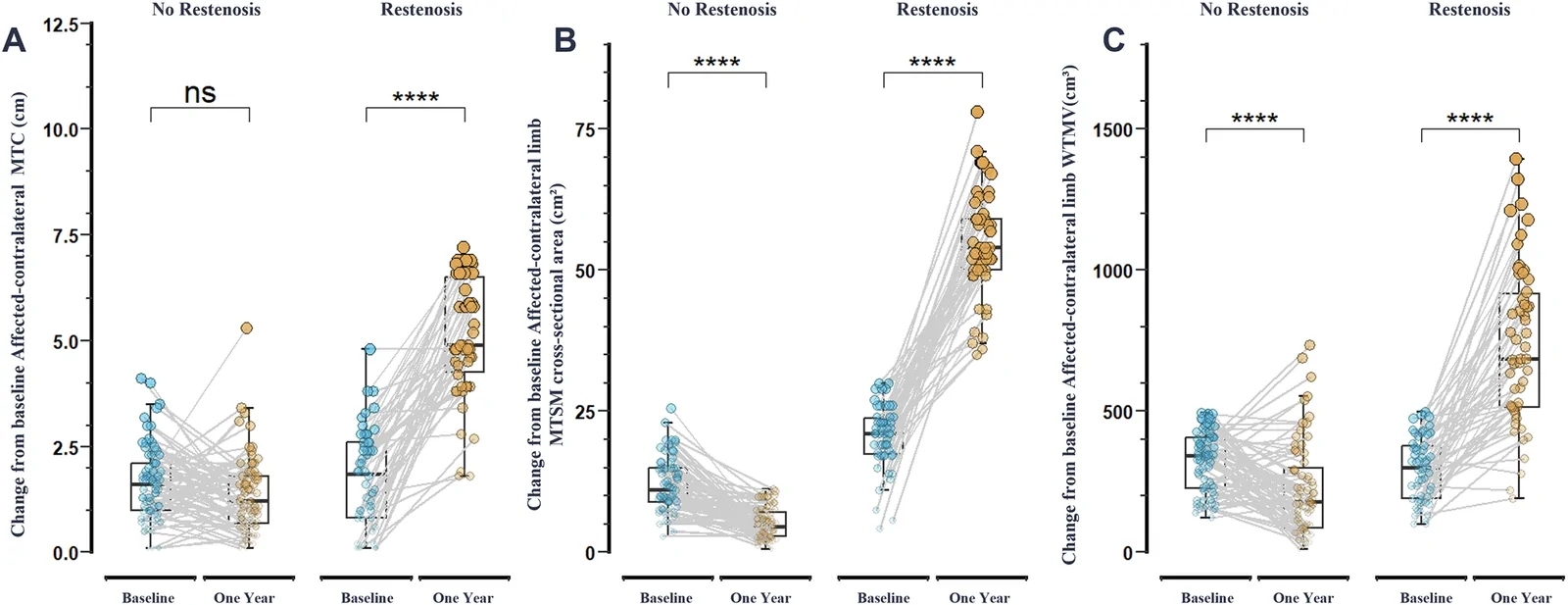
Longitudinal contralateral-controlled thigh muscle changes by restenosis status. **A** Affected minus contralateral mid-thigh circumference at baseline and 1 year in patients without restenosis and with restenosis. **B** Affected minus contralateral mid-thigh skeletal muscle cross-sectional area at baseline and 1 year. **C** Affected minus contralateral whole-thigh muscle volume at baseline and 1 year. Dots represent individual patients and gray lines connect paired measurements; boxplots summarize the distribution within each subgroup

At 1 year, mid-thigh circumference was lower in the restenosis group for both the affected limb (30.79 ± 8.22 cm vs 43.24 ± 4.87 cm, *p* < 0.01) and the contralateral limb (35.88 ± 8.74 cm vs 44.16 ± 4.62 cm, *p* < 0.01), with a larger affected-minus-contralateral difference (5.09 ± 1.41 cm vs 1.36 ± 0.92 cm, *p* < 0.01). Mid-thigh skeletal muscle area at 1 year was higher in the restenosis group for the affected limb (215.18 ± 51.69 cm² vs 194.58 ± 49.58 cm², *p* = 0.03) and the contralateral limb (269.90 ± 56.35 cm² vs 188.04 ± 47.29 cm², *p* < 0.01), yielding a larger affected-minus-contralateral difference (54.72 ± 9.27 cm² vs 5.13 ± 2.79 cm², *p* < 0.01). Contralateral whole-thigh muscle volume was also higher in the restenosis group (5166.33 ± 1847.85 cm³ vs 4068.68 ± 1314.75 cm³, *p* < 0.01), and the affected-minus-contralateral difference was larger (732.24 ± 284.03 cm³ vs 221.89 ± 166.38 cm³, *p* < 0.01). Figure 3 shows the changes from baseline in contralateral-controlled metrics differed between groups, including affected-minus-contralateral mid-thigh circumference (−7.86 ± 13.83 vs −0.37 ± 1.90, *p* < 0.01), mid-thigh skeletal muscle area (−2.16 ± 2.25 vs 0.49 ± 0.31, *p* < 0.01), and whole-thigh muscle volume (−1.93 ± 1.86 vs 0.22 ± 0.59, *p* < 0.01).

### Univariable and multivariable logistic regression analyses for restenosis

In univariable analyses, restenosis was significantly associated with longer lesion length, higher baseline stenosis degree, more advanced Fontaine stage, longer disease duration, more complex TASC II classification (C/D), lower affected-limb ankle-brachial index, baseline affected-minus-contralateral mid-thigh skeletal muscle cross-sectional area difference, several 1-year CTA muscle measures, and the 1-year change in affected-minus-contralateral mid-thigh circumference (Table 2). In the multivariable modeling, lesion length (OR, 1.55; 95% CI: 1.21–2.61; *p* < 0.01) and the baseline affected-minus-contralateral mid-thigh skeletal muscle area difference (OR, 1.51; 95% CI: 1.10–1.95; *p* = 0.01) were independent predictors in the predictive model (Model 2), which used only baseline variables. In the full explanatory model (Model 3), additional independent predictors included 1-year affected-minus-contralateral whole-thigh muscle volume (OR, 0.25; 95% CI: 0.19–0.75; *p* < 0.01) and contralateral-controlled longitudinal changes in mid-thigh circumference (OR, 1.74; 95% CI: 1.21–2.55; *p* < 0.01) and mid-thigh skeletal muscle area (OR, 1.51; 95% CI: 1.18–2.01; *p* = 0.03). The final explanatory model included 5 predictors for 162 events, yielding an events-per-variable ratio of 32.4, suggesting low risk of overfitting.

**Table 2:** Univariate and multivariate logistic regression analyses for restenosis

Characteristics	Univariate		Multivariate	
	OR (95% CI)	*p*-value	OR (95% CI)	*p*-value
Age (years)	0.99 (0.94–1.04)	0.7		
Gender (male, %)	0.86 (0.43–1.75)	0.68		
Height (cm)	1.12 (0.92–1.37)	0.25		
Weight (kg)	1.04 (0.98–1.09)	0.21		
BMI	0.83 (0.66–1.04)	0.1		
Hypertension (%)	0.96 (0.48–1.95)	0.92		
Dyslipidemia (%)	1.12 (0.55–2.31)	0.75		
Diabetes (%)	0.79 (0.36–1.75)	0.56		
Smoking history (%)	1.12 (0.55–2.31)	0.75		
Treatment (%)	1.04 (0.51–2.11)	0.92		
Length of the lesion (cm)	1.18 (1.02–1.54)	< 0.01	1.55 (1.21–2.61)	< 0.01
Disease duration (per month)	1.07 (1.04–1.10)	< 0.01	1.04 (1.01–1.07)	0.02
Fontaine stage III–IV (vs II)	2.61 (1.71–3.99)	< 0.01	1.82 (1.15–2.88)	0.01
Baseline stenosis degree (per %)	1.05 (1.03–1.07)	< 0.01	1.03 (1.01–1.05)	0.02
TASC C/D (vs A/B)	3.07 (2.00–4.71)	< 0.01	1.45 (0.91–2.31)	0.12
Affected limb ABI	0.62 (0.51–0.75)	< 0.01	0.47 (0.21–0.55)	0.28
Baseline				
Affected limb MTC (cm)	0.81 (0.72–0.91)	0.99		
Contralateral limb MTC (cm)	0.93 (0.88–0.98)	0.93		
Affected-contralateral MTC (cm)	1.13 (0.79–1.60)	0.04	1.27 (0.87–1.82)	0.5
Affected limb MTSM cross-sectional area (cm²)	1.00 (1.00–1.01)	0.47		
Contralateral limb MTSM cross-sectional area (cm²)	0.97 (0.91–1.02)			
Affected-contralateral limb MTSM cross-sectional area (cm²)	1.11 (1.03–1.19)	< 0.01	1.51 (1.10–1.95)	0.01
Affected limb WTMV (cm³)	0.98 (0.92–1.24)	0.47		
Contralateral limb WTMV (cm³)	0.99 (0.87–1.23)	0.35		
Affected-contralateral limb WTMV (cm³)	1.00 (0.99–1.00)	0.15		
1-year follow-up				
Affected limb MTC (cm)	0.83 (0.77–0.89)	< 0.01	0.31 (0.10–0.58)	0.19
Contralateral limb MTC (cm)	1.00 (0.93–1.07)	0.96		
Affected-contralateral MTC (cm)	0.90 (0.83–0.97)	< 0.01	0.97 (0.91–1.03)	0.34
Affected limb MTSM cross-sectional area (cm²)	1.02 (1.01–1.53)	< 0.01	2.09 (1.12–3.51)	0.02
Contralateral limb MTSM cross-sectional area (cm²)	1.03 (0.79–1.41)	0.57		
Affected-contralateral limb MTSM cross-sectional area (cm²)	2.25 (1.51–3.01)	0.29		
Affected limb WTMV (cm³)	1.21 (1.10–1.32)	0.08		
Contralateral limb WTMV (cm³)	0.91 (0.75–1.10)	0.96		
Affected-contralateral limb WTMV (cm³)	0.60 (0.45–0.79)	< 0.01	0.25 (0.19–0.75)	< 0.01
Change from baseline				
Affected-contralateral MTC (cm)	1.52 (1.15–1.79)	< 0.01	1.74 (1.21–2.55)	< 0.01
Affected-contralateral limb MTSM cross-sectional area (cm²)	1.53 (1.12–1.78)	0.08	1.51 (1.18–2.01)	0.03
Affected-contralateral limb WTMV (cm³)	1.06 (0.92–1.87)	< 0.01	1.53 (1.12–2.78)	0.08

*MTC* mid-thigh circumference, *MTSK* mid-thigh skeletal muscle, *WTMV* whole-thigh muscle volume

### Sensitivity analysis adjusting for disease severity

In the prespecified sensitivity analysis (Supplementary Table 1), when baseline stenosis degree, Fontaine stage, disease duration, and TASC II classification were added as covariates alongside lesion length, the contralateral-controlled muscle asymmetry metric remained independently associated with restenosis (OR 1.43, 95% CI: 1.08–1.89, *p* = 0.01). Baseline stenosis degree (OR 1.03 per %, 95% CI: 1.01–1.05, *p* = 0.02), Fontaine stage III–IV (OR 1.82, 95% CI: 1.15–2.88, *p* = 0.01), and disease duration (OR 1.04 per month, 95% CI: 1.01–1.07, *p* = 0.02) were also independently associated with restenosis. TASC C/D classification did not retain independent significance after adjustment for the other severity variables (OR 1.45, 95% CI: 0.91–2.31, *p* = 0.12), likely reflecting collinearity with stenosis degree and lesion length. Adding the muscle metric to a model containing only severity variables increased AUC from 0.76 to 0.79 (DeLong *p* = 0.04), supporting incremental discrimination.

### Model discrimination and clinical utility

**Fig. 4:**
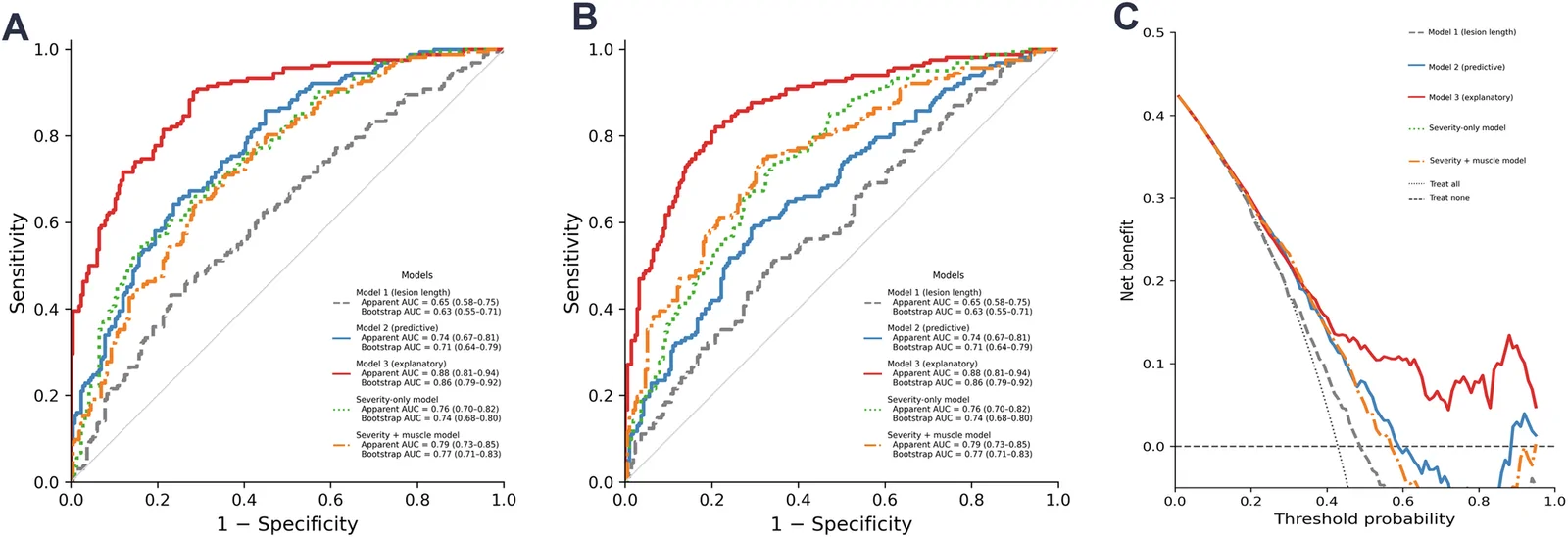
Model discrimination and clinical utility for prediction of restenosis. **A** Receiver operating characteristic curves showing apparent discrimination for three prespecified logistic regression models: Model 1 (lesion length alone); Model 2 (Predictive Model: Model 1 + baseline affected-contralateral MTSK area); Model 3 (Explanatory Model: Model 2 + all 1-year and longitudinal muscle metrics). **B** Bootstrap-validated receiver operating characteristic curves with corresponding AUCs and 95% confidence intervals. **C** Decision curve analysis comparing net benefit across threshold probabilities for selecting patients for confirmatory DSA

Across the prespecified models, discrimination for 1-year restenosis improved stepwise. In the apparent analysis (Fig. 4A), AUC increased from 0.65 (95% CI: 0.58–0.75) for Model 1 (lesion length) to 0.74 (95% CI: 0.67–0.81) for the predictive Model 2 (Model 1 + baseline muscle difference), and to 0.88 (95% CI: 0.81–0.94) for the explanatory Model 3 (Model 2 + 1-year and longitudinal muscle metrics). For comparison, the severity-only model (lesion length + baseline stenosis degree + Fontaine stage + disease duration + TASC II) achieved an apparent AUC of 0.76 (95% CI: 0.70–0.82); adding the contralateral-controlled muscle metric to this model raised the AUC to 0.79 (95% CI: 0.73–0.85), with a DeLong *p*-value of 0.04 for the AUC difference. After bootstrap validation (Fig. 4B), AUCs were 0.63 (95% CI: 0.55–0.71) for Model 1, 0.71 (95% CI: 0.64–0.79) for Model 2, 0.86 (95% CI: 0.79–0.92) for Model 3, 0.74 (95% CI: 0.68–0.80) for the severity-only model, and 0.77 (95% CI: 0.71–0.83) for the severity + muscle model. Decision curve analysis (Fig. 4C) showed that Model 3 provided the highest net benefit across clinically relevant threshold probabilities, while the severity + muscle model also offered net benefit superior to Model 1, Model 2, and the “treat-all”/“treat-none” reference strategies.

The consistency between imaging assessments and clinical reference standards was good, with agreement coefficients of 0.852 for Reader 1 and 0.901 for Reader 2 (both *p* < 0.05). The inter- and intra-observer reliability for quantitative CT imaging measurements is summarized in Table 3.

**Table 3:** Inter- and intra-observer reliability of CT imaging features

Characteristics	Inter-observer ICC (95% CI)	Intra-observer ICC (95% CI)
Baseline		
Affected limb MTC (cm)	0.87 (0.85, 0.92)	0.89 (0.84, 0.93)
Contralateral limb MTC (cm)	0.89 (0.81, 0.92)	0.85 (0.81, 0.92)
Affected limb MTSM cross-sectional area (cm²)	0.9 (0.88, 0.95)	0.87 (0.84, 0.9)
Contralateral limb MTSM cross-sectional area (cm²)	0.97 (0.93, 0.99)	0.96 (0.94, 0.98)
Affected limb WTMV (cm³)	0.94 (0.91, 0.97)	0.96 (0.93, 0.99)
Contralateral limb WTMV (cm³)	0.96 (0.93, 0.98)	0.93 (0.91, 0.97)
1-year follow-up		
Affected limb MTC (cm)	0.91 (0.86, 0.96)	0.92 (0.88, 0.96)
Contralateral limb MTC (cm)	0.96 (0.91, 0.99)	0.93 (0.89, 0.96)
Affected limb MTSM cross-sectional area (cm²)	0.95 (0.92, 0.98)	0.96 (0.93, 0.98)
Contralateral limb MTSM cross-sectional area (cm²)	0.95 (0.91, 0.97)	0.97 (0.94, 0.98)
Affected limb WTMV (cm³)	0.92 (0.89, 0.99)	0.94 (0.91, 0.98)
Contralateral limb WTMV (cm³)	0.96 (0.93, 0.98)	0.98 (0.92, 0.98)

## Discussion

In this retrospective study, we identified contralateral-controlled thigh muscle metrics on CTA as novel predictors of 1-year restenosis following endovascular therapy for unilateral PAD. Our primary predictive model, using only baseline clinical and imaging data, demonstrated that the difference in muscle area between the affected and healthy limbs is an independent predictor of future restenosis, even after adjustment for baseline stenosis severity, Fontaine stage, and disease duration. An explanatory model incorporating longitudinal muscle changes showed that patients who developed angiographic restenosis had greater baseline muscle atrophy and showed worsening muscle composition over 1 year. Taken together, muscle asymmetry appears to add prognostic information to conventional arterial lesion characteristics.

This addition warrants careful interpretation, because muscle atrophy and arterial disease severity are not biologically independent. Chronic ischemia drives myocyte loss, fatty replacement, and reduced capillary density in the affected limb, so a more severely diseased limb tends to have both worse arteries and worse muscles. Our contralateral-controlled metrics therefore likely function as integrative biomarkers of cumulative ischemic burden rather than as causally independent predictors. What the contralateral-controlled design offers, however, is a partial separation of limb-specific end-organ effects from body-wide factors. Even after adjustment for lesion length, baseline stenosis degree, Fontaine stage, disease duration, and TASC II classification, muscle asymmetry retained an independent association with restenosis (OR 1.43, 95% CI: 1.08–1.89, *p* = 0.01), and adding the muscle metric to the severity-only model increased the AUC from 0.76 to 0.79 (DeLong *p* = 0.04). The incremental signal is modest but statistically meaningful, and is most plausibly explained by muscle asymmetry reflecting cumulative ischemic tissue damage and local inflammatory burden that stenosis degree or clinical staging alone do not fully capture.

Our results extend the growing body of evidence linking adverse muscle composition to outcomes in PAD [15, 16]. Prior studies using CT have shown that low skeletal muscle density is associated with increased mortality and major limb events in PAD [17, 18]. McDermott and colleagues reported that lower calf muscle density and greater fat content were associated with higher all-cause mortality in PAD, highlighting muscle quality as a prognostic marker [19]. Lin et al demonstrated that low muscle density independently predicted amputation risk in PAD [20]. Our study builds on these insights by showing that muscle quantity metrics can also be prognostically useful when considered in a relative context. In fact, whereas prior work noted that absolute muscle volume alone had limited predictive value for events, we found that a greater side-to-side muscle area deficit is a strong risk factor for restenosis [21]. This suggests that asymmetric limb muscle loss carries prognostic information not captured by raw muscle size.

It is noteworthy that few studies have specifically examined revascularization durability in relation to muscle health [22]. Few studies have examined revascularization durability in relation to muscle health. To our knowledge, our study is one of the first to demonstrate that preoperative muscle atrophy and post-procedure muscle remodeling are linked to arterial restenosis outcomes. This finding aligns with the concept of sarcopenia as an indicator of frailty and poor tissue reserve. Taniguchi et al observed that sarcopenic patients with chronic limb-threatening ischemia had worse limb salvage rates, and a 2021 review concluded that sarcopenia is a negative prognostic factor in PAD [23, 24]. Our results are consistent with these observations, now showing that muscle deficits portend not only limb loss risk but even the failure of endovascular interventions. We also confirmed known predictors of restenosis reported in the literature [25]. In our cohort, longer lesion length was associated with higher restenosis odds. This concurs with prior clinical experience that extensive femoropopliteal lesions carry greater risk of restenosis, due to a larger plaque burden and longer arterial segments exposed to stenting trauma. Interestingly, we found contralateral muscle measures also differed between groups, patients who re-stenosed tended to have smaller baseline muscle mass overall, implying a degree of systemic muscle depletion or worse fitness. This resonates with the notion that PAD patients with generalized sarcopenia or frailty may fare worse after interventions. In summary, our study adds new evidence that muscle imaging biomarkers can stratify risk of device failure, complementing prior research on muscle metrics and PAD prognosis.

The link between thigh muscle metrics and restenosis risk likely reflects both the severity of ischemic injury and the biological interplay between muscle health and vascular healing [26, 27]. In-stent restenosis (ISR) is fundamentally an aberrant healing response to vessel injury, involving early inflammatory cell infiltration, mid-phase vascular smooth muscle cell (VSMC) migration, and late extracellular matrix remodeling in the treated segment [28]. Patients with advanced PAD often have chronically ischemic muscles in the affected limb, characterized by myocyte loss, fatty replacement, and reduced capillary density [29]. This chronic muscle pathology can create a pro-inflammatory, pro-fibrotic local environment [30]. Ischemic muscle tissue releases various cytokines and growth factor in response to hypoxia and injury. While such factors aim to promote angiogenesis or muscle regeneration, they may also stimulate intimal hyperplasia and neointimal growth in adjacent arteries [31]. In essence, a limb with severe muscle loss may be a limb with high levels of ischemia-induced inflammatory signaling, which in turn exacerbates re-stenotic processes [32]. This could explain why baseline muscle atrophy was an independent predictor of stent failure in our study. Moreover, poor muscle health might be a surrogate for systemic factors that impede vascular healing. Sarcopenia is associated with chronic inflammation and endothelial dysfunction [33]. Patients with low muscle mass often have higher circulating inflammatory markers and reduced anabolic hormones, conditions that can impair arterial repair and favor scar tissue formation [34]. Conversely, when revascularization is successful, improved perfusion may halt muscle degeneration. The role of structured physical exercise, a cornerstone of PAD management, is an important related factor not measured in our study. Exercise could potentially mitigate muscle atrophy and improve vascular health, thereby influencing restenosis risk, and represents a key area for future research. It is crucial, however, to interpret these proposed mechanisms as hypothesis-generating, as our observational study design does not allow for the establishment of causality.

## Limitations

This study presents several limitations. First, its single-center, retrospective design and the exclusion of patients with bilateral PAD required to utilize the contralateral limb as an internal control, introduce selection bias and limit generalizability to the broader PAD population. Second, requiring baseline and 1-year follow-up imaging may have skewed the cohort toward specific disease severities. Furthermore, because DSA was performed only when clinically indicated rather than as universal angiographic screening, detection bias is likely. In particular, asymptomatic restenosis may have been missed, and the observed restenosis rate should therefore be interpreted as the rate of clinically driven, DSA-confirmed restenosis rather than the true incidence of all angiographic restenosis at 1 year. In addition, duplex ultrasound was not routinely performed in every patient, which further limits the complete detection of subclinical restenosis. These limitations should be addressed in future prospective studies using predefined imaging surveillance with routine duplex ultrasound and/or CTA, with DSA reserved for confirmatory assessment when clinically appropriate. Third, despite sensitivity analyses adjusting for clinical severity, muscle atrophy may partially surrogate cumulative ischemic burden, leaving potential residual confounding. Using contralateral limbs as controls may have also underestimated true between-limb effects due to subclinical atherosclerosis (< 50% stenosis). Finally, our methodology was restricted in scope. We evaluated thigh muscle area and volume but excluded calf musculature, anatomical stenosis stratification, and muscle density (due to multicollinearity). Statistically, while bootstrap validation was performed, optimism-corrected calibration metrics and a full correlation matrix were omitted. External validation in prospective, multicenter cohorts is necessary.

## Conclusion

Contralateral-controlled thigh muscle metrics on routine CTA may help refine risk stratification for restenosis without additional imaging cost. A predictive model using only baseline data identified high-risk patients who may benefit from targeted surveillance, while the explanatory model showed a consistent relationship between longitudinal muscle change and vascular outcomes. The independent association of muscle asymmetry with restenosis after adjustment for disease severity warrants further validation in multicenter prospective cohorts.

## Supplementary information

ELECTRONIC SUPPLEMENTARY MATERIAL

## Data Availability

The datasets generated or analyzed during the study are available from the corresponding author upon reasonable request.

## Abbreviations

AUC: Area under the curve
CTA: Computed tomography angiography
DSA: Digital subtraction angiography
MTC: Mid-thigh circumference
MTSK: Mid-thigh skeletal muscle
PAD: Peripheral artery disease
WTMV: Whole-thigh muscle volume
