# Supplementary material for: Contralateral-controlled thigh muscle biomarkers on CT angiography for prediction of 1-year restenosis after endovascular therapy for unilateral peripheral artery disease
Source: Insights Imaging. 2026 Oct 7;17:241. doi: 10.1186/s13244-026-02407-0 (PMC13646228; doi:10.1186/s13244-026-02407-0)

Contralateral-Controlled Thigh Muscle Biomarkers on CT Angiography for Prediction of 1-Year Restenosis after Endovascular Therapy for Unilateral Peripheral Artery Disease

ELECTRONIC SUPPLEMENTARY MATERIAL

Insights Imaging (2026) Lin S, Wang X, Pan Y, Zhang L, Sun J, Chen B

Supplementary Table 1. Sensitivity analysis: multivariable logistic regression for 1-year restenosis adjusting for disease severity measures.

Predictor	Univariable OR (95% CI)	P	Severity-adjusted OR (95% CI)*	P
**Disease severity covariates**				
Lesion length (per cm)	1.18 (1.02–1.54)	<0.01	1.41 (1.12–1.78)	<0.01
Baseline stenosis degree (per %)	1.05 (1.03–1.07)	<0.01	1.03 (1.01–1.05)	0.02
Fontaine stage III–IV (vs II)	2.61 (1.71–3.99)	<0.01	1.82 (1.15–2.88)	0.01
Disease duration (per month)	1.07 (1.04–1.10)	<0.01	1.04 (1.01–1.07)	0.02
TASC C/D (vs A/B)	3.07 (2.00–4.71)	<0.01	1.45 (0.91–2.31)	0.12
**Contralateral-controlled muscle biomarker**				
**Baseline side-to-side MTSK difference (per cm²)**	1.11 (1.03–1.19)	<0.01	1.43 (1.08–1.89)	0.01

The severity-adjusted model included lesion length, baseline stenosis degree, Fontaine stage, disease duration, TASC II classification, and baseline side-to-side MTSK difference. Discrimination: AUC of the severity-adjusted muscle model = 0.79 (95% CI: 0.73–0.85), compared with AUC = 0.76 (95% CI: 0.70–0.82) for the disease-severity-only model (without muscle metric); **ΔAUC = 0.03 (DeLong P = 0.04)**, supporting that contralateral-controlled muscle asymmetry adds incremental discrimination beyond established disease severity measures.*Events per variable in the severity-adjusted model: 162 events / 6 predictors = 27.0.**CI, confidence interval; MTSK, mid-thigh skeletal muscle cross-sectional area; OR, odds ratio; TASC, Trans-Atlantic Inter-Society Consensus.*

Supplementary

Case 1 A 73-year-old male admitted for lower extremity arteriosclerosis obliterans. Computed tomography shows occlusion of the left lower extremity artery (A). (B)

Muscle segmentation using threshold segmentation (from -20 Hounsfield units to 150 Hounsfield units). (C) Volume rendering technique shows preoperative muscle distribution. (D) Subsequently, the patient underwent stent placement.

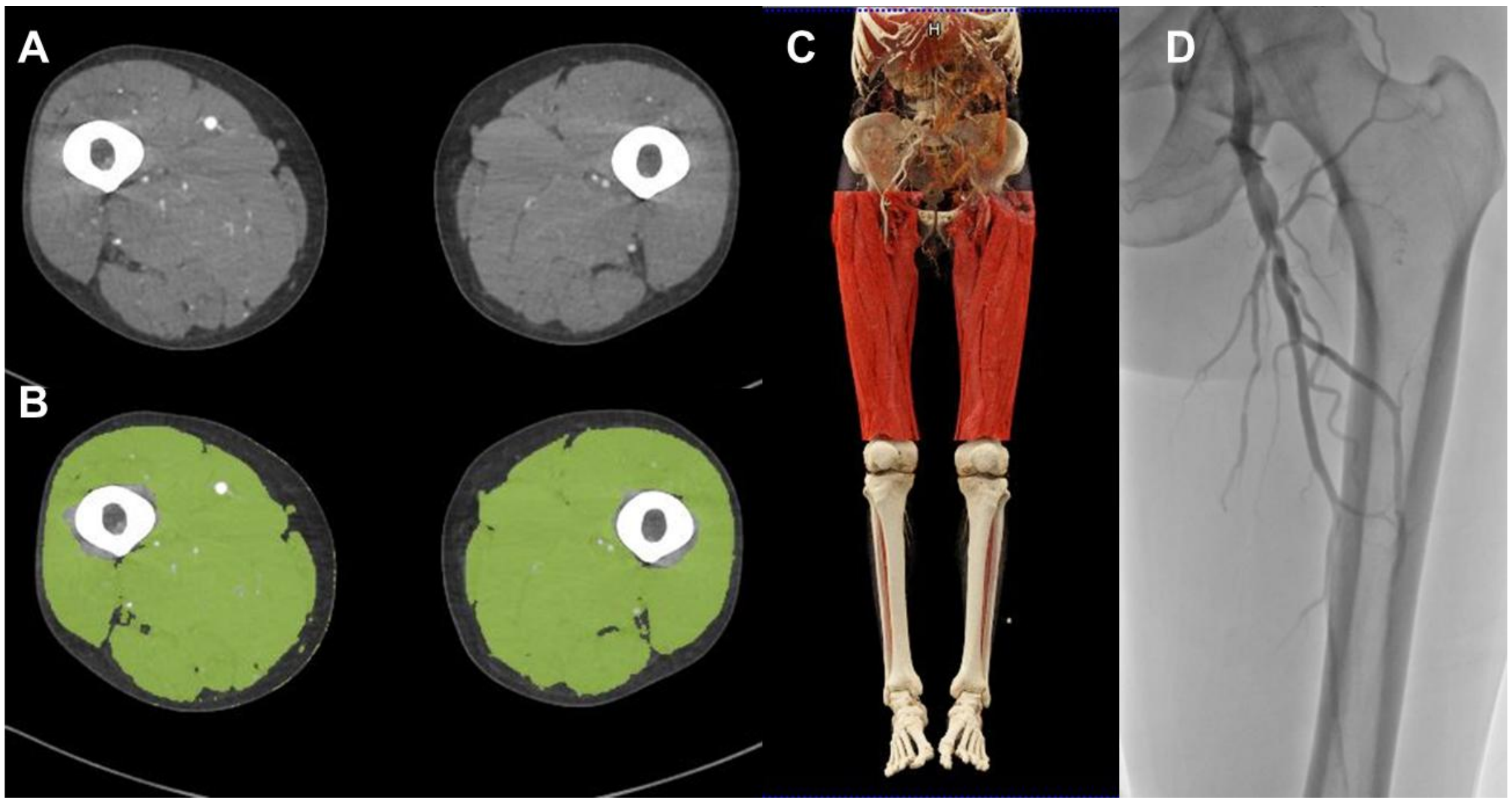

One year later, the patient underwent contrast-enhanced computed tomography angiography of both lower limbs. The computed tomography angiography showed (A) in-stent occlusion of the left lower extremity artery. (B) Muscle segmentation using threshold segmentation (from -20 Hounsfield units to 150 Hounsfield units). (C) Volume rendering technique shows muscle distribution after stent placement. (D, E) Subsequently, the patient underwent open surgical incision and stent recanalization.

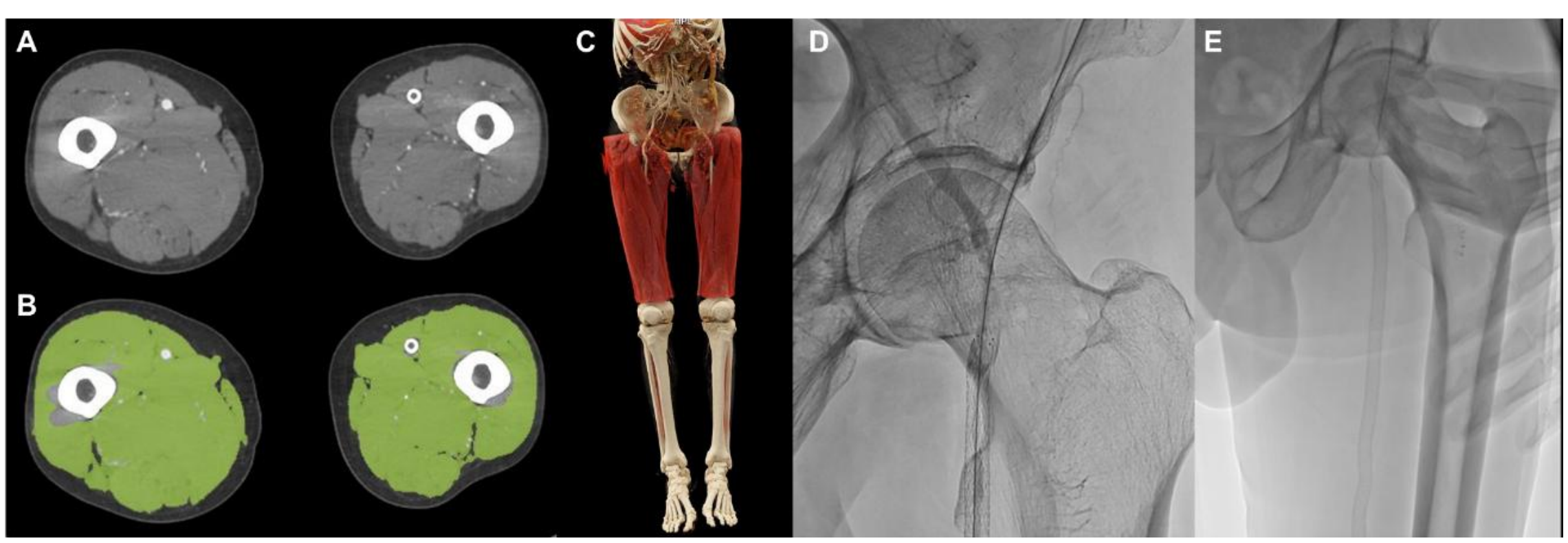

Supplementary

Case 2 An 86-year-old female admitted for evaluation due to claudication. (A) Digital subtraction angiography shows occlusion of the femoral artery segment in the right lower extremity. (B) Curved planar reformation of computed tomography angiography shows occlusion of the femoral artery segment in the right lower extremity. (C, D) Axial images with threshold segmentation (from -29 Hounsfield units to 150 Hounsfield units) for muscle segmentation.

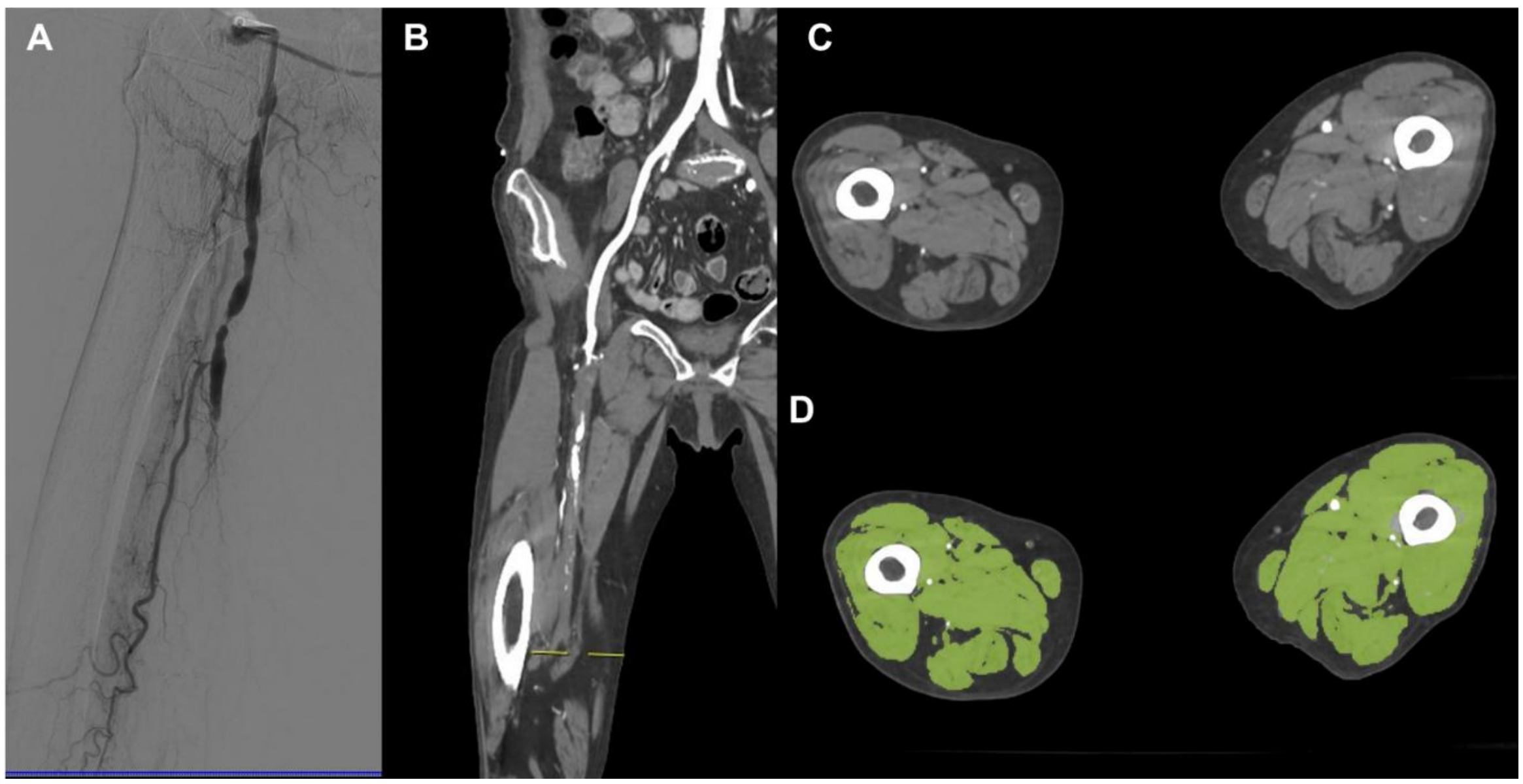

(A) Digital subtraction angiography and (B) curved planar reformation of computed tomography angiography show in-stent occlusion of the femoral artery segment in the right lower extremity, as well as occlusion of the femoral artery segment of the right lower extremity artery. (C, D) Axial images with threshold segmentation (from -20 Hounsfield units to 150 Hounsfield units) for muscle segmentation. (E) Volume

rendering technique shows muscle distribution after stent placement.

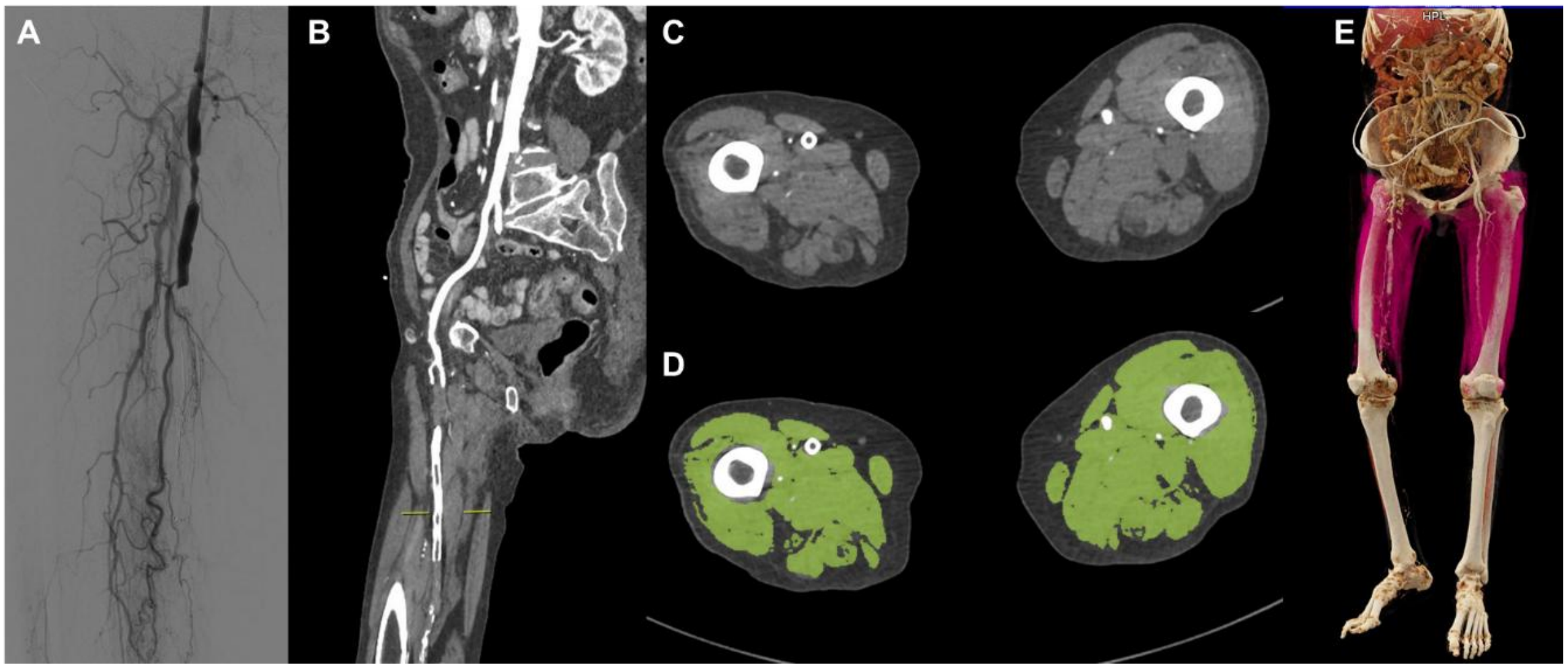

Supplement: Supplementary file 1 [file 13244_2026_2407_MOESM1_ESM.pdf]
